# Multi-center screening of the Pathogen Box collection for schistosomiasis drug discovery

**DOI:** 10.1186/s13071-019-3747-6

**Published:** 2019-10-22

**Authors:** Martina Maccesi, Pedro H. N. Aguiar, Valérian Pasche, Melody Padilla, Brian M. Suzuki, Sandro Montefusco, Ruben Abagyan, Jennifer Keiser, Marina M. Mourão, Conor R. Caffrey

**Affiliations:** 1 Center for Discovery and Innovation in Parasitic Diseases, Skaggs School of Pharmacy and Pharmaceutical Sciences, University of California, San Diego, La Jolla, CA USA; 2 Laboratório de Helmintologia e Malacologia Médica, René Rachou Institute, FIOCRUZ, Belo Horizonte, Minas Gerais Brazil; 3 Department of Medical Parasitology and Infection Biology, Swiss Tropical and Public Health Institute, P.O. Box, 4002 Basel, Switzerland; 4 University of Basel, P.O. Box, 4003 Basel, Switzerland; 5 Present Address: Telethon Institute of Genetics and Medicine (TIGEM), Pozzuoli, NA Italy

**Keywords:** *Schistosoma*, Schistosomiasis, Drug discovery, Phenotypic screen, Pathogen Box, MMV

## Abstract

**Background:**

Over the past five years, as a public service to encourage and accelerate drug discovery for diseases of poverty, the Medicines for Malaria Venture (MMV) has released box sets of 400 compounds named the Malaria, Pathogen and Stasis Boxes. Here, we screened the Pathogen Box against the post-infective larvae (schistosomula) of *Schistosoma mansoni* using assays particular to the three contributing institutions, namely, the University of California San Diego (UCSD) in the USA, the Swiss Tropical and Public Health Institute (Swiss TPH) in Switzerland, and the Fundação Oswaldo Cruz (FIOCRUZ) in Brazil. With the same set of compounds, the goal was to determine the degree of inter-assay variability and identify a core set of active compounds common to all three assays. New drugs for schistosomiasis would be welcome given that current treatment and control strategies rely on chemotherapy with just one drug, praziquantel.

**Methods:**

Both the UCSD and Swiss TPH assays utilize daily observational scoring methodologies over 72 h, whereas the FIOCRUZ assay employs XTT (2,3-bis(2-methoxy-4-nitro-5-sulfophenyl)-5-[(phenylamino)carbonyl]-2H-tetrazolium hydroxide) at 72 h to measure viability as a function of NAD^+^/NADH redox state. Raw and transformed data arising from each assay were assembled for comparative analysis.

**Results:**

For the UCSD and Swiss TPH assays, there was strong concordance of at least 87% in identifying active and inactive compounds on one or more of the three days. When all three assays were compared at 72 h, concordance remained a robust 74%. Further, robust Pearsonʼs correlations (0.48–0.68) were measured between the assays. Of those actives at 72 h, the UCSD, Swiss TPH and FIOCRUZ assays identified 86, 103 and 66 compounds, respectively, of which 35 were common. Assay idiosyncrasies included the identification of unique compounds, the differential ability to identify known antischistosomal compounds and the concept that compounds of interest might include those that *increase* metabolic activity above baseline.

**Conclusions:**

The inter-assay data generated were in good agreement, including with previously reported data. A common set of antischistosomal molecules for further exploration has been identified
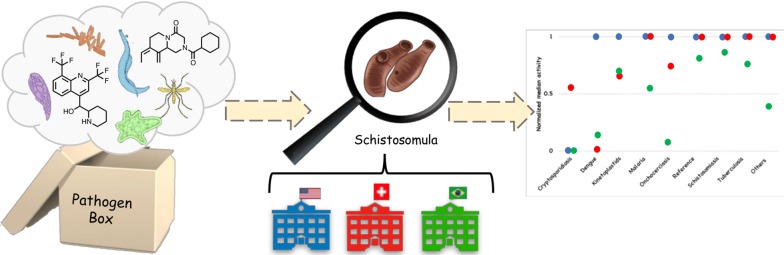
.

## Background

Schistosomiasis is a parasitic disease caused by trematodes of the genus *Schistosoma*. The disease is endemic in 78 countries and preventive chemotherapy of at least 206 million people was necessary in 2016 [1]. Of concern for such a prevalent disease, treatment and control relies on mass chemotherapy with just one drug, praziquantel (PZQ) [2, 3]. PZQ is safe, active against all species of *Schistosoma* and is affordable, or distributed free of charge. However, the increasing distribution of what is very often a sub-curative drug may encourage the emergence of PZQ-tolerant, or worse, PZQ-resistant parasites [3–5], an alarming prospect in the absence of any back-up drug.

Unlike the case for other global infectious diseases of poverty, schistosomiasis has lacked the prioritization necessary to establish trans-national, public-private drug development programmes that support the synthesis of small molecule chemical libraries, high throughput screening systems and the discovery expertise to identify and advance compounds to the clinic. Allied to this is the greater difficulty, logistics and costs needed to maintain and handle the schistosome parasite compared to the self-replicative single-celled organisms like malaria or the kinetoplastid parasites that are relatively easy to culture and amenable to automated liquid handling robotics. Accordingly, schistosome drug discovery remains fragmented across the academic sector with its more limited resources.

In the last decade, the realization that the ever-increasing distribution of just one drug to sustain the management of schistosomiasis is untenable has motivated the development of a number of phenotypic screening paradigms to identify new small molecules of interest. Many of these have coalesced around the application of *Schistosoma mansoni* schistosomula (post-infective larvae) [6–8]. These can be harvested in their tens of thousands, are reasonably amenable to liquid handling and have been used as the entry-point to screening and triaging the larger ‘industrial-scale’ of small molecule collections for subsequent tests against the more limiting adult parasite which, although directly responsible for disease morbidity *via* their eggs, can only be obtained in small numbers and from small vertebrate animals. A plethora of schistosomula assays with different read-outs has been developed [6, 8–11].

Over the same period, the Medicines for Malaria Venture (MMV) has made available to the drug discovery community a number of box sets each containing 400 well-annotated small molecules that have been validated in various disease contexts. These include the Malaria Box [12], Pathogen Box [13], Stasis Box and, most recently, the Pandemic Box. All, bar the last box, have been screened against schistosomes in culture/*in vivo* and the data made publicly available [12, 14–16]. Because the general interpretability of data arising from the phenotypic screening of schistosomula with the various MMV-supplied boxes (and other small molecules) may have been influenced by the particular assay methodology employed, we took the opportunity to perform an inter-institutional phenotypic evaluation of the Pathogen Box. The study involved teams from the University of California San Diego (UCSD), the Swiss Tropical and Public Health Institute (Swiss TPH), and the Fundação Oswaldo Cruz (FIOCRUZ). With the same chemical matter in hand, the goals were to identify a core set of active anti-schistosomal compounds of interest for future preclinical pursuit and highlight some distinct features of each particular assay.

## Methods

### Compounds

The Pathogen Box collection contains 400 compounds distributed in five 96-well plates and dissolved in 10 μl of pure dimethyl sulfoxide (DMSO) at a concentration of 10 mM. Plates were shipped on dry ice to each of the three institutions by the MMV. Supporting information for the compound collection can be found at https://www.mmv.org/mmv-open.

### Life-cycle of *S. mansoni* at UCSD and preparation of schistosomula

The NMRI isolate of *S. mansoni* is maintained by passage through *Biomphalaria glabrata* snails (NMRI line) and 3–5 week-old, female Golden Syrian hamsters (Envigo, Placentia, CA, USA) as intermediate and definite hosts, respectively [6, 17]. The acquisition, preparation and *in vitro* maintenance of post-infective larvae (schistosomula) are described as follows. Briefly, infected patent snails were induced to shed infective larvae (cercariae) under a lamp for up to 2 h. Cercariae were concentrated over ice for up to 1 h and then mechanically transformed to schistosomula *via* tail-shearing through a 22 gauge micro-emulsifying needle attached to two 10 ml syringes (passaged back and forth 12 times) [18]. Schistosomula were washed in ice-chilled Basch Medium 169 [19] (containing 100 U/ml penicillin and 100 mg/ml streptomycin). Schistosomula were collected and washed three times in the same medium and kept on ice for a maximum of 1 h before distribution into assay plates containing compounds.

### *In vitro* phenotypic assay of schistosomula at UCSD

The assay was performed as described [6, 20, 21]. Essentially, stock Pathogen Box compounds were diluted in DMSO to a 2 mM concentration and 1 µl manually spotted into transparent, U-bottomed 96-well assay plates (Costar 3367). Chilled Basch Medium 169 (100 µl; containing 100 U/ml penicillin, 100 mg/ml streptomycin and 4% heat-inactivated FBS (Corning Mediatech, New York, USA) was then added to mix the compound and this was followed by another 100 µl of medium containing 40–50 schistosomula. The final concentration of compound and DMSO was 10 µM and 0.5%, respectively. Each compound was tested in duplicate and solvent controls, containing 0.5% DMSO only, were placed in columns 1 and 12 of each plate. Assay plates were placed into plastic boxes humidified with wet tissue and these were incubated at 37 °C in a 5% CO_2_ environment.

Phenotypic changes in the schistosomula were visually recorded at 24, 48 and 72 h using a Zeiss Axiovert A1 inverted microscope (100×) as described [6, 22–24]. Briefly, simple descriptors that describe the effects of compounds on the parasites (changes in shape, motility and density) are employed. To allow for comparisons of compound activity, each descriptor is awarded a ‘severity score’ of 1 and these are added up to a maximum score of 4. Evidence of degeneracy or death is awarded the maximum score of 4 (raw data shown in Additional file 1: Table S1). Scores were averaged across the duplicate wells.

### Life-cycle of *S. mansoni* at the Swiss TPH and preparation of schistosomula

The procedures to maintain the *S. manson*i (Liberian strain) life-cycle, harvest cercariae and obtain schistosomula for *in vitro* testing are described elsewhere [8]. As detailed previously, the cercariae were mechanically transformed into schistosomula using two syringes connected by a Luer-Lok® connector (B. Braun Melsungen AG, Melsungen, Germany) [8, 18, 25]. Schistosomula were adjusted to a concentration of 50 units/100 µl and incubated for at least 12 h at 37 °C in a 5% CO_2_ environment before use [14].

### *In vitro* phenotypic assay of schistosomula at the Swiss TPH

On the day of the first drug assay, the 10 mM stock solutions of the Pathogen Box compounds were diluted (1:10) in Medium 199 (Gibco, USA) supplemented with 5% fetal calf serum (FCS; Bioconcept AG, Allschwil, Switzerland) and an antibiotic cocktail developed previously [26]. The drug plates were then stored at − 20 °C upon further use. In flat-bottom 96-well plates, each compound was tested in duplicate at a concentration of 10 µM. Every test plate included two negative controls wells containing 0.1% DMSO. Assay plates were incubated at 37 °C in a 5% CO_2_ environment.

Phenotypic changes were visually recorded (10× magnification) at 24, 48 and 72 h using a previously described scoring method [8, 27]. Briefly, each well was scored using a quarter point descending scale from 3 to 0. The maximum score of 3 was assigned to wells containing schistosomula with normal movement and for which no morphological changes were apparent. A score of 2 was assigned when the overall movement in the well was reduced (or abnormally increased) and when morphological changes became apparent, i.e. increase in granularity, swelling, etc. A score of 1 was applied when almost no movement and more severe morphological alterations were observed. Finally, a score of zero indicated worm death (no movement and a complete loss of integrity). The antischistosomal effect was expressed as a fraction of the average test scores compared to the average score of the negative control wells using Microsoft Excel as (1 − average(test)/average(control)) [8]. For the assay to be considered valid, a minimal average score of 2 was required in the control wells.

### Life-cycle of *S. mansoni* at FIOCRUZ and preparation of schistosomula

Cercariae of *S. mansoni* (LE strain) were harvested from *B. glabrata* (Barreiro strain). Schistosomula were obtained by mechanical transformation of cercariae using a protocol adapted from that previously described [19]. Briefly, cercariae were distributed into 50 ml conical tubes and allowed to settle on ice for 60 min. Cercariae were concentrated by centrifugation at 1000×*g* for 3 min at 4 °C, followed by resuspension in Medium 199 (without phenol red; Cat. # M3769, Sigma-Aldrich, Buchs, Switzerland) supplemented with 100 U/ml penicillin and 100 μg/ml streptomycin (GIBCO, Thermo Fisher Scientific, Waltham, MA, USA). Cercariae were mechanically transformed by passing them four times through a 22-gauge syringe needle followed by 10 cycles of washing and sedimentation. Schistosomula were distributed in flat-bottom 96-well culture plates at 200 parasites/well in a final volume of 195 µl, and incubated at 37 °C and 5% CO_2_ for 24 h in the same medium supplemented with 2% heat-inactivated FBS (GIBCO, Thermo Fisher Scientific).

### *In vitro* phenotypic assay of schistosomula at FIOCRUZ

XTT (sodium-2,3-bis-[2-methoxy-4-nitro-5-sulfophenyl]-2H–tetrazolium-5-carboxanilide) was used as an indicator of schistosomula metabolic activity by the reduction of the yellow tetrazolium salt XTT to an orange formazan product [28, 29]. XTT was dissolved in Medium 199 without FBS to prepare a 1 mg/ml solution and the electron coupling reagent, N-methyl dibenzopyrazine methyl sulfate (PMS; Cat. # P9625, Sigma-Aldrich) was dissolved at 0.383 mg/ml in PBS. Both the XTT and PMS solutions were filtered through a 0.2 µm pore size membrane and stored at – 20 °C until use. Pathogen Box compounds were diluted to 400 µM from a 10 mM stock concentration in Medium 199 (without phenol red) in V-bottom 96-well assay plates. Afterwards, 5 µl of the diluted compounds were added to the culture plates that contained 200 parasites/well in 200 µl (10 µM final concentration of compound). Incubations were maintained for 48 h at 37 °C and 5% CO_2_. The XTT labeling mixture was prepared by mixing the XTT and PMS solutions in a 50:1 ratio and 40 µl of the mixture was added to each well of the assay plate. The incubations were continued for a further 24 h at 37 °C and 5% CO_2_ and the absorbance at 450 nm (reference wavelength of 690 nm) was determined using a SpectraMax M5 microplate reader (Molecular Devices, San Jose, CA, USA). Positive and negative controls comprised heat-killed parasites and parasites incubated in the presence of 0.1% DMSO, respectively. All compounds were tested in duplicate in two independent experiments, totaling four wells for each compound. Schistosomula viability was determined using absorbance values applied to the following equation:

$$\% \;{\text{Viability}} = \frac{{\left( {{\text{Sample}} - {\text{Positive}}\;{\text{control}}} \right)}}{{\left( {{\text{Negative}}\;{\text{control}} - {\text{Positive}}\;{\text{control}}} \right)}} \times 100$$

where “Sample” is the absorbance measured in each well containing parasites tested with compounds, and “Negative control” and “Positive control” represent the average absorbance measured in each of the respective controls.

### Data analysis

The phenotypic scores generated by UCSD and Swiss TPH, and the percentage viability data generated by FIOCRUZ are presented in Additional file 1: Table S2. Active compounds were identified as follows: an UCSD assay score of ≥ 2, a Swiss TPH drug effect of ≥ 0.5 and a viability of ≤ 50% for the FIOCRUZ assay. The data from the three assays were also mathematically transformed (below) to obtain activity values within the same range and thus facilitate direct comparisons (Additional file 1: Table S3). Before running the transformation, the raw data were inspected. For some of the compounds tested in the Swiss TPH assay, negative viability values were obtained when the scores of the test compounds surpassed the scores in the control wells. These were converted to zero to indicate inactivity. Similarly, in the FIOCRUZ assay, compounds inducing an increase over the 100% baseline viability of the DMSO controls were not included in the data transformation process. However, some of these compounds that increased activity over baseline were also associated with antischistosomal activity in at least one of the other two assays and these are discussed below. Finally, because the scoring methods applied were opposite to one another, i.e. for the UCSD and Swiss TPH, the greater the value the more active the compound, whereas for FIOCRUZ, the reverse was the case, the raw data values were inverted to be consistent across all three groups. The data were then transformed in a span range from 0 (inactive) to 1 (most active) using the following equation:

$${\text{Transformed}}\;{\text{data}} = \frac{{\left( {{\text{x}} - {\text{median}}} \right)}}{{\left( {\hbox{max} \left( {\text{x}} \right) - {\text{median}}} \right)}}$$

The median was selected as the threshold below which all the compounds were considered inactive. We employed the median instead of the minimum value of each series given that the majority of compounds were inactive. Data analysis was performed using Microsoft Office Excel and Molsoft ICM software [30]. For the transformed data, the Pearsonʼs correlation was calculated between pairs of data sets. The value for the F-distribution was also calculated to determine whether the correlation values occurred by chance or not. A low F-distribution value indicates that there is a low probability that the correlation between activity data occurred by chance. Finally, data for the normalized median activity score at 72 h were generated in R (version 3.6.0) using the *ggplot2* package (version 3.2.0) [31].

## Results and discussion

### Active and inactive Pathogen Box compounds identified among the three institutions

*Schistosoma mansoni* schistosomula were screened with the 400 constituent compounds of the MMV Pathogen Box by three institution-specific assays developed by UCSD, the Swiss TPH and FIOCRUZ. The data arising from each assay were then assembled for comparative analysis (Additional file 1). Our study is relevant as, to date, a plethora of schistosomula screening assays employing different methodologies (e.g. ATP and NAD(P) metabolic indicators, DNA intercalation agents such as propidium iodide, and visual- or automated image-based systems) and readouts (e.g. percentage death, EC_50_ value or phenotypic score), have been developed such that the general interpretability of data may be constrained by the particular assay employed [9–11, 24, 28, 32–34].

The main questions to address were the extent to which the data generated are assay-specific and which compounds can be considered common actives or non-actives independent of the assay employed. Table 1 indicates the degree of concordance in the number of actives and non-actives identified across the three assays. The complete dataset is presented in Additional file 1: Table S2.

**Table 1:** Degree of concordance in the number of actives and non-actives identified in the Pathogen Box between the UCSD (U), Swiss TPH (S) and FIOCRUZ (F) assays

Time (h)/Comparison	U *vs* S	U *vs* F	S *vs* F	U *vs* S *vs* F
24	**87** (349)	na	na	na
48	**87** (348)	na	na	na
72	**87** (347)	**83** (331)	**78** (312)	**74** (295)

*Notes*: For the times indicated, the percentage (in bold) and number of compounds (in parentheses) that were identified as active or non-active between the institutions are indicated. Comparisons between UCSD and the Swiss TPH were run on a daily basis, whereas comparisons across all three groups were possible at the 72-h time point when the XTT assay (FIOCRUZ) was completed and the data analyzed

*Abbreviation*: na, not applicable

For the UCSD and Swiss TPH assays, which employ visual interpretations of anti-schistosomal activity as a function of time, there was strong concordance of 87% in identifying compounds as being either active or inactive at 24, 48 and 72 h (Table 1). When comparing the UCSD and Swiss TPH assays with the FIOCRUZ metabolic-based XTT assay at the 72 h time point, agreement remained high, i.e. 83% and 78% between FIOCRUZ and UCSD, and FIOCRUZ and SWISS TPH, respectively. When data of all three groups were compared at 72 h, the concordance was lower but still a robust 74%. This lower score might be attributable to the assay differences and parasite strains. In particular, the single metric XTT assay used by FIOCRUZ measures the oxidation of NAD(P)H as an indicator of viability [29] whereas visual interpretations of activity used by UCSD and the Swiss TPH are more holistic by identifying changes in features such as size, shape, color and motility, relative to DMSO controls.

**Fig. 1:**
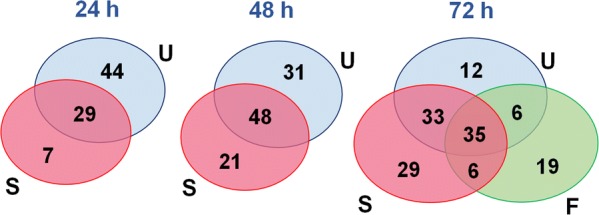
Active Pathogen Box compounds identified by the three institutions. Venn diagram indicating the number of unique or shared actives identified by the three assays. Comparisons across all three groups were only possible at the 72-h time point when the XTT assay (FIOCRUZ) was completed and the data analyzed. U (blue), S (magenta) and F (green) represent UCSD, Swiss TPH and FIOCRUZ, respectively

Because both the UCSD and Swiss TPH assays employed daily observations at 24, 48 and 72 h, it was possible to note some trends for those compounds declared as active. The number of unique actives identified by UCSD decreased over time [44, 31 and 18 compounds at 24, 48 and 72 h (not counting the FIOCRUZ component at 72 h), respectively] (Fig 1; Additional file 1: Table S4). This was partly due to transiently active compounds, whereby changes noted at 24 h were no longer obvious at the later time points. Such compounds have been noted before [6, 35] and involve relatively mild changes in shape and/or motility, and with scores no greater than 2. The second trend was the increasing proportion of actives that was shared with the Swiss TPH assay as a function of time such that by 72 h, 68 active compounds were shared. These shared actives generally involved more progressive, irreversible and degenerative phenotypes (Additional file 1: Table S2). Nonetheless, at 72 h, UCSD had 18 active compounds (of which 10 registered strong scores ≥ 3), that were not identified by the Swiss TPH assay: these may be due to differences in the assay design and/or interpretation, or the parasite strain (NMRI at UCSD and Liberian at the Swiss TPH).

In contrast to the UCSD assay, the number of unique Swiss TPH actives increased as a function of time from seven at 24 h to 35 at 72 h (not counting the FIOCRUZ component at 72 h; Fig. 1; Additional file 1: Table S4). This may be due to differences in the screen assessment methods or parasite strains. The scoring system used by UCSD is based on a range of phenotypic descriptors that encompass shape and color, in addition to motility. Although these parameters are taken into account in the Swiss TPH scoring scale, the emphasis is on motility. A closer examination of the 35 compounds identified as active by the Swiss TPH at 72 h indicates that 27 had borderline or modest scores of between 0.5 and 0.65 (Additional file 1: Table S4). In contrast, and as noted above, those actives that were shared between Swiss TPH and UCSD at 72 h and the earlier time points tended to be more potent, i.e. scores of ≥ 0.75 for Swiss TPH and ≥ 3 for UCSD.

At 72 h, the FIOCRUZ assay identified 66 actives, less than the 86 and 103 actives captured by the UCSD and Swiss TPH assays, respectively (Fig. 1; Additional file 1: Table S2). Of these FIOCRUZ actives, 35 were shared with both other assays (termed ‘core actives;’ Additional file 1: Table S5), an additional 12 actives were shared with either UCSD (six) or Swiss TPH (six), and 19 were unique. Of the 35 core actives, the majority (29) were strongly active (≤ 30% viability) in the FIOCRUZ assay and were also registered as strong actives in the other two assays (scores ≥ 3 and ≥ 0.75 for UCSD and Swiss TPH, respectively) at 72 h (Additional file 1: Table S5).

As a single-metric assay using the dye XTT, the FIOCRUZ assay is designed to specifically measure metabolic viability via NAD(P)H turnover, whereas the other two assays are based on the observational appraisals of phenotypic effects. Thus, it is anticipatable that the number of actives captured overall by the XTT assay would be less than the observation-based methods as depicted in Fig. 1. Nonetheless, the XTT assay has a robust 74% concordance with the other two assays and, in terms of automation, scalability and stringency, is particularly suitable for high-throughput formats [28]. One possibility for improving the active capture rate of the XTT assay is to introduce a time component; however, this would require more parasites and compound, hence the current decision to use a single 72 h time point.

The finding of 19 unique XTT hits is intriguing and, apart from the possible influence of strain, the result may indicate the assay’s particular ability to identify compounds that decrease the metabolic fitness of the parasite yet are beyond visual detection in either of the other two assays. Indeed, 12 of the 19 compounds decreased viability to less than 25% of the control value (Additional file 1: Table S4). Based on a PubChem search, we note that four of the 19 XTT-active compounds inhibit *Plasmodium falciparum* l-lactate dehydrogenase, which employs NADP(H) to catalyze the interconversion of lactate and pyruvate. Interference of this metabolic pathway in the schistosome would likely be detected by the XTT assay as it could interfere with the parasite’s dehydrogenase activity.

Finally, the discussion above related to the XTT assay is based on measuring *a decrease* in metabolic activity from the 100% baseline. When including those compounds that *increase* activity above baseline, arbitrarily > 130%, an additional 34 compounds are identified, and of these, nine compounds are also active in at least one of the other assays (Additional file 1: Table S2). The association between increased metabolic rate and anti-schistosomal activity demonstrated here has not been noted before but is worthy of consideration going forward.

To allow for direct comparisons of the data arising from the three assays, the raw activity data were transformed and ranked on a scale of 0 to 1 from least to most active (Additional file 1: Table S3). The transformed data were used to calculate the Pearsonʼs correlation and the respective value of the F-distribution at the different time points. Robust correlation values of 0.61, 0.63 and 0.68 were recorded at 24, 48 and 72 h, respectively, between the UCSD and Swiss TPH assay data, indicating that most of the active compounds demonstrated comparable activity. As might be anticipated with the XTT assay, the Pearsonʼs correlation values were more moderate when compared to either the UCSD or Swiss TPH assays at 72 h (0.46 and 0.50, respectively).

### Activity of reference compounds and consistency with previous data

**Fig. 2:**
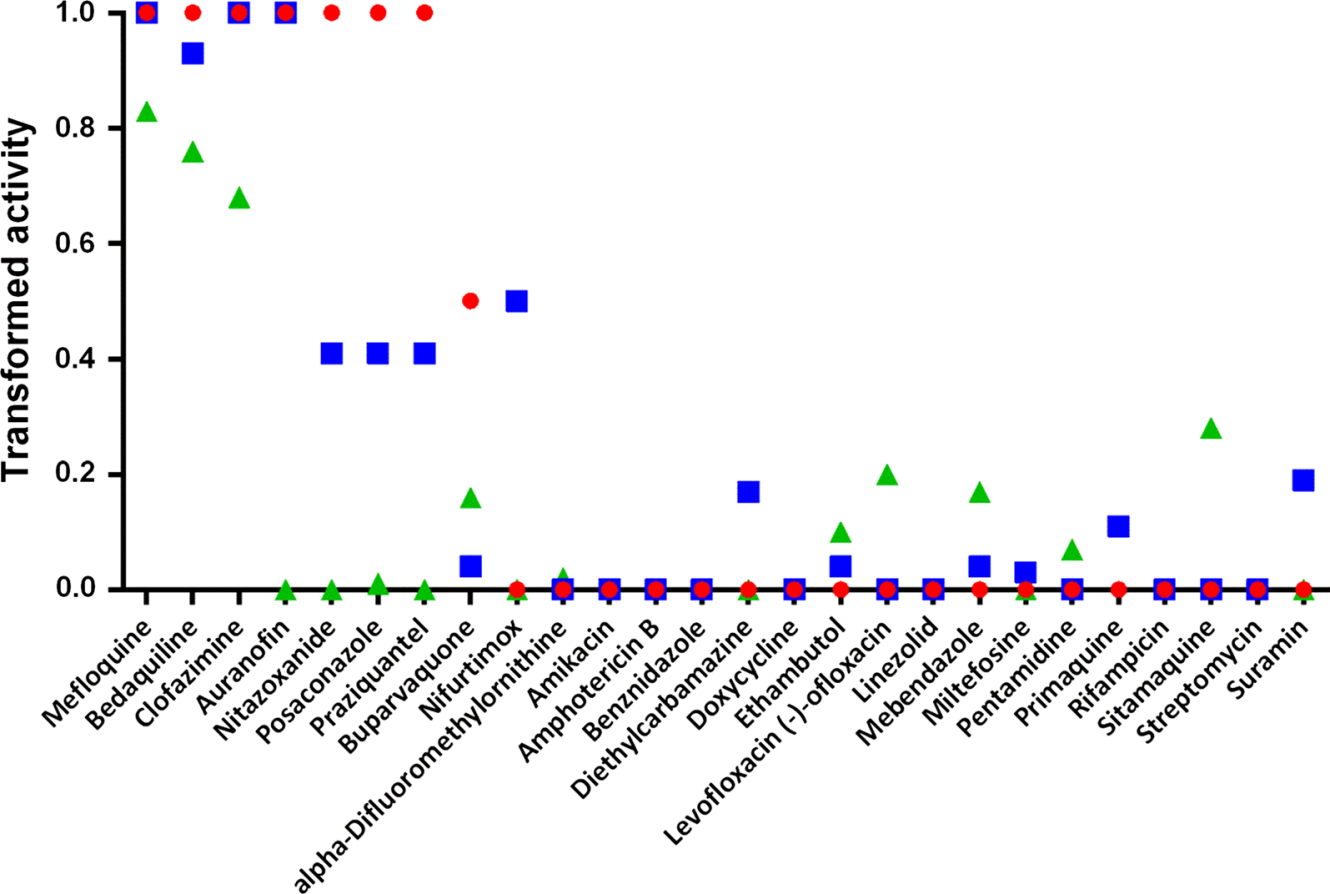
Activity of 26 reference compounds in the Pathogen Box as measured by each assay. Data were transformed on a scale of 0 to 1 to allow for direct comparisons of the three datasets at the 72-h time point. Data for UCSD (red circles), Swiss TPH (blue squares) and FIOCRUZ (green triangles) are indicated

Included in the Pathogen Box are 26 reference compounds, i.e. compounds known to be active against and/or marketed as drugs for various microbial diseases. As shown in Fig. 2 (also Additional file 1: Table S6), eight of the 26 reference compounds were identified as active in the UCSD and Swiss TPH assays at the 72-h time point. Among these were drugs that had been previously identified as antischistosomal, namely clofazimine [36], mefloquine [37], auranofin [38, 39] and nitazoxanide [6]. Both groups also had unique actives, namely, buparvaquone (UCSD) and nifurtimox (Swiss TPH). In the Swiss TPH assay, by contrast, PZQ generated a transformed score of 0.4 (raw score of 0.56), i.e. borderline active. The discrepancy in the activity between the two assays in detecting PZQ may be due to parasite strain or scoring methodology.

The FIOCRUZ assay returned three actives among the 26 reference compounds, namely, mefloquine, bedaquilline and clofazimine: each of these was strongly active in the other two assays. PZQ was not active in the XTT assay. In this context, it is pertinent to note that a previously employed CellTiter-Glo assay methodology which utilizes ATP as the metabolic readout did not identify PZQ or oxamniquine, an older marketed drug for treatment of schistosomiasis mansoni, as active against schistosomula [32]. This contrasts with the morphological and motility derangements induced by PZQ as detected here and previously [6, 40, 41]. We speculate that metabolic-based assays like the XTT assay which are designed to measure viability in single cells, are prone to missing important anti-schistosomal chemistries that may not affect cellular vitality/viability *per se*, but have mechanisms of action of particular consequence to complex metazoans, e.g. dysregulation of the neuromuscular system.

Interestingly, among the reference compounds that were inactive in all three assays was miltefosine, which was shown to be active against *S. mansoni* miracidia and cercariae *in vitro*, as well as against eggs, and adult worms *in vitro* and *in vivo* [42–44]. The mechanism of action of miltefosine may depend on the interplay between parasite and host. Thus, El-Faham and colleagues [45] demonstrated that treating parasites with miltefosine enhances serological recognition of defined adult worm surface antigens. The absence of activity here may suggest a stage-specific mode of action, i.e. lacking activity against schistosomula.

Finally, of note, is the inter-assay consistency in the number of shared actives identified here and previously during the assembly of the Pathogen Box in 2015. Specifically, of 13 compounds declared by the MMV as ‘schistosomiasis active’, nine had been identified as active in the Swiss TPH assay against schistosomula after 72 h (https://www.mmv.org/sites/default/files/uploads/docs/mmv_open/Pathogen_Box_Activity_Biological_Data_Smiles.xlsx). In the present setting and at the same time point, six of those nine actives were confirmed by the Swiss TPH assay and corroborated by the UCSD assay, with the latter identifying one additional compound (Table 2; Additional file 1: Table S7). Lastly, the FIOCRUZ assay identified five of the same six active compounds, plus three additional actives, one of which was shared with UCSD. Overall, therefore, there was solid cross-assay consistency and recall in confirming actives among those compounds previously designated by the MMV as ‘schistosomiasis active’.

**Table 2:** Actives among the three assays for 13 compounds that had been designated earlier as ‘schistosomiasis active’ by the MMV

	UCSD			Swiss TPH			FIOCRUZ (mean ± SD)
**MMV ID/Time point (h)**	**24**	**48**	**72**	**24**	**48**	**72**	**72**
MMV688761	**4**	**4**	**4**	**1.00**	**1.00**	**1.00** ^a^	**−** **8.0** ± 2.5
MMV688763	**4**	**4**	**4**	**1.00**	**1.00**	**1.00** ^a^	**−** **5.8** ± 4.9
MMV688762	**4**	**4**	**4**	**1.00**	**1.00**	**1.00** ^a^	**−** **3.4** ± 3.7
MMV688768	**3**	**4**	**4**	**1.00**	**1.00**	**1.00** ^a^	**6.4** ± 3.3
MMV688178	0	0	**2**	**0.60**	**1.00**	**1.00** ^a^	**3.1** ± 8.5
MMV676382	**4**	**4**	**4**	0.45	**0.68**	**0.94** ^a^	61.7 ± 11.2
MMV688270	**2**	**4**	**4**	0.05	0.00	0.11^a^	**39.0** ± 24.6
MMV688766	0	0	0	0.15	0.17	0.19	**0.1** ± 0.4
MMV688313	0	**2**	0	0.10	0.17	0.28^a^	**14.7** ± 1.7
MMV688771	1	**2**	0	0.05	0.10	0.16	62.2 ± 7.1
MMV676536	0	0	0	0.10	0.14	0.11^a^	75.5 ± 36.1
MMV688552	0	0	0	0.15	0.17	0.31	117.5 ± 14.6
MMV1198433	0	0	0	0.10	0.06	0.00	86.0 ± 19.9

^a^Compounds that the Swiss TPH had previously determined as ‘schistosomiasis active’ *vs* schistosomula at 72 h during the assembly of the Pathogen Box. Active compounds are delineated in bold typeface

### Actives identified as a function of disease set

**Fig. 3:**
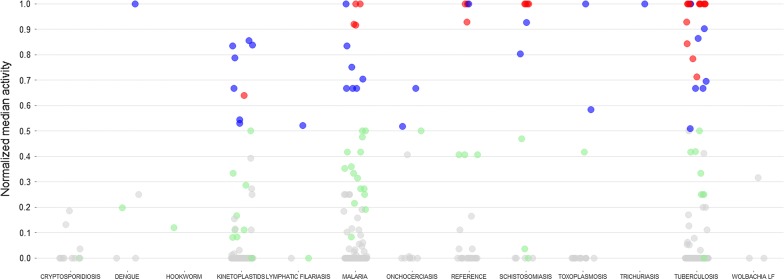
Antischistosomal activity as a function of disease set in the Pathogen Box. The Pathogen Box contains compounds for which activity against various micro-organisms had been previously determined during the assembly of the collection (disease sets). For each compound (represented as a circle), the normalized median activity score at 72 h was calculated and plotted as a function of disease. A grey circle indicates that a median activity score of < 0.5 was attributed by all of the screening centers, whereas green, blue and red circles indicate that a score ≥ 0.5 was attributed by one, two or all three centers, respectively

Apart from the strong representation from the MMV ‘schistosomiasis actives’ among the identified hits (Table 2), there were also representations from most of the other 11 sets designated by the MMV as being active against a particular disease (Fig. 3; Additional file 1: Table S8). As examples, one of four dengue compounds, MMV688543, was active, i.e. normalized median activity ≥ 0.5, in both the UCSD and Swiss TPH assays. Among the 70 kinetoplastid compounds, eight (MMV658988, MMV676602, MMV688273, MMV688283, MMV688372, MMV689244, MMV690027 and MMV690102) were active in at least two of the assays. Similar findings were made for 11 of the 125 malaria compounds (MMV001059, MMV020391, MMV020623, MMV022029, MMV022478, MMV023233, MMV023985, MMV024114, MMV024406, MMV663250 and MMV676881), two of the 11 onchocerciasis compounds (MMV671636 and MMV676063), one of the three filariasis compounds (MMV687775) and two of 15 toxoplasmosis compounds (MMV688364 and MMV688417). Among the 116 tuberculosis compounds, 21 were active in two or more assays. Finally, among the 11 cryptosporidiosis, one hookworm, and three *Wolbachia* compound sets, actives in two or more assays were not identified (Fig. 3; Additional file 1: Table S8).

## Conclusions

In this study, the Pathogen Box compound set was phenotypically screened with *S. mansoni* schistosomula using assays originating from three different institutions, namely, UCSD, the Swiss TPH and FIOCRUZ. The study’s goals were to assess the degree of inter-assay variability and identify a core set of compounds that are active across the three assays performed. Among the inherent variables were parasite strain, culture medium, whether or not the parasites were pre-incubated prior to addition of compounds and the detection system employed. In this context, field isolates of *S. mansoni* with differential susceptibilities PZQ are known [46] and the choice of culture medium can influence the viability of schistosomula [7, 8, 17]. Evaluating the possible influences of these variables on the data, however, was not a goal of the current study. Our data show that concordance in identifying actives and non-actives was greatest between the UCSD and the Swiss TPH which employ visual assessments of phenotypic changes; however, the single-metric XTT assay employed by FIOCRUZ maintained the concordance among all groups at a robust 74%. Each assay identified unique actives, including the finding with the XTT assay that a chemically-induced increase in baseline metabolic rate is associated with antischistosomal activity. Thus, it could be argued that the best strategy to increasing the identification of active compounds is to combine an observation-based approach with the single-metric XTT assay, assuming that the resources necessary are not limiting. Overall, a common core set of 35 active compounds was identified which could be considered for further investigation. In this context, the activity of 24 out of the 35 core actives has already been tested against adult *S. mansoni* [15] with 13 compounds being active on both developmental stages (Additional file 1: Table S9). These particular hits provide starting points for further optimization.

## Supplementary information

**Additional file 1: Table S1.** Descriptors and associated severity scores applied by UCSD. **Table S2.** Concordance between the 3 groups for active and inactive compounds. **Table S3.** Data transformation. **Table S4.** Unique hits per group. **Table S5**. Active compounds shared by all three groups (‘core actives’). **Table S6**. Transformed activity of the reference compounds. **Table S7**. Comparisons with data previously generated for the same compounds. **Table S8.** Transformed activity by disease set. **Table S9.** Core active compounds compared to activity measured previously by the Swiss TPH against adult *S. mansoni*.

## Data Availability

The data supporting the conclusions of this article are included within the article.

## Abbreviations

ATP: adenosine triphosphate
DMSO: dimethyl sulfoxide
DNA: deoxyribonucleic acid
EC50: half maximal effective concentration
FBS: fetal bovine serum
FCS: fetal calf serum
FIOCRUZ: Fundação Oswaldo Cruz
MMV: Medicines for Malaria Venture
NAD(P): nicotinamide adenine dinucleotide (phosphate)
NMRI: Naval Medical Research Institute
PBS: phosphate-buffered saline
PMS: N-methyl dibenzopyrazine methyl sulfate
PZQ: praziquantel
Swiss TPH: Swiss Tropical and Public Health Institute
UCSD: University of California San Diego
XTT: sodium-2,3- bis-[2-methoxy-4-nitro-5-sulfophenyl]-2H–tetrazolium-5-carboxanilide
